# When the Cover Burns: Behavioral and Morphological Responses of Western Fence Lizards (*Sceloporus occidentalis*) to Increased Openness in Post‐Fire Environments

**DOI:** 10.1002/ece3.73673

**Published:** 2026-06-22

**Authors:** Elmer E. Gutierrez, Breanna J. Putman

**Affiliations:** ^1^ California State University, San Bernardino San Bernardino California USA; ^2^ California State University, Long Beach Long Beach California USA

**Keywords:** avian visual model, crypsis, fire ecology, luminance, microhabitat selection, predation risk

## Abstract

Wildfires are increasing in frequency and intensity due to human activity, yet the behavioral and morphological responses of animals to post‐fire stressors remain poorly understood. We linked increased canopy openness in recently burned areas with a greater number of predator attacks on clay models of Western Fence Lizards ( *Sceloporus occidentalis* ). We also examined whether lizards that recently experienced a fire exhibited morphological shifts in dorsal darkness to better match the darkened substrates of burned environments and whether they shifted perch use to enhance background matching on substrates that reduce conspicuousness in these altered environments. Lizard dorsal darkness and background matching increased with substrate darkness (i.e., they were better camouflaged against burned substrates), but populations from burned habitats were not overall darker in coloration than those from unburned habitats. This was likely due to a preference for using unburned wooden perches, resulting in an average dorsal darkness comparable to lizards in unburned sites. Despite increased predator attacks in recently burned areas, lizards may not consistently adopt behaviors that enhance background matching in post‐fire landscapes. This study provides valuable insights into the behaviors of animals affected by fires and highlights potential trade‐offs that could affect reproduction or survival.

## Graphical abstract

We found that Western Fence Lizards experience higher detectability in burned areas but do not consistently darken or adjust perch use to enhance background matching, despite showing improved camouflage on darker substrates. These results suggest that lizards may face behavioral trade‐offs in post‐fire landscapes that could influence success.

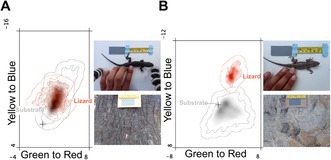

## 1 Introduction

Wildfires are a natural phenomenon that have shaped ecosystems for millions of years (Scott 2000), promoting key ecological processes and driving adaptations across species (Baum and Sharber 2012; Pausas 2015a, 2015b; Pausas and Keeley 2014; Wagenius et al. 2020; McLauchlan et al. 2020). However, the frequency and intensity of fires are increasing rapidly due to anthropogenic activities and climate change (Balch et al. 2017; Westerling and Bryant 2008). These human‐driven fires disrupt native ecosystems and threaten species already at risk of extinction (Kelly et al. 2020; Garcês and Pires 2023; Santos, Hradsky, et al. 2022). Yet, wildlife persist and reproduce in frequently burned habitats, presumably by altering behaviors and morphologies in response to fire‐induced selection pressures. One major consequence of wildfire is large‐scale changes in vegetation structure (Denno et al. 2005; Foster et al. 2017; Li et al. 2022), which may alter predator–prey dynamics. Reduced vegetation cover increases visibility and detection distances, potentially favoring predators (Leahy et al. 2016; Cherry et al. 2017) by increasing vulnerability of prey that rely on structural cover for protection (Doherty et al. 2015; Horn et al. 2012). Predators may also be drawn to recently burned areas, where prey are more exposed (Hovick et al. 2017). Although in some cases prey may benefit from enhanced visibility to detect predators (Jaffe and Isbell 2009; Cherry et al. 2018), changes in predation risk (high or low) should result in changes to antipredator traits. If prey vulnerability increases after wildfires, prey animals may need to heighten antipredator defenses in burned habitats.

One of the first lines of defense of prey is to avoid detection by predators (Lima and Dill 1990; Putman et al. 2015). Animals may select microhabitats that reduce their likelihood of detection while balancing access to resources needed for reproduction and survival (Dupuch et al. 2009; Korpimaki et al. 1996; Glen et al. 2010). Animals may use areas that provide optimal background matching, allowing their body coloration or patterns to blend with the surrounding environment. For example, Jacky dragons ( *Amphibolurus muricatus* ) preferentially use complex backgrounds that improve camouflage, increasing their chances of avoiding detection by predators and/or prey (Salisbury and Peters 2019). To successfully become cryptic, animals must have a color pattern matching their background in size, color, and brightness distribution (Endler 1978). In post‐burned areas, melanistic (black) coloration may be favored to camouflage against the charred background environment. Such variation has been documented in diverse taxa including invertebrates (Forsman et al. 2011; Rocha et al. 2015; de Alcantara Viana et al. 2024), mammals (Guthrie 1967; Potash et al. 2020), and reptiles (De Miranda et al. 2022). Thus, a darker coloration and/or enhanced background matching may be a crucial adaptation to avoid detection by roving predators in recently burned areas where vegetation cover is reduced. Yet, to date, little research has focused on how animals specifically alter antipredator traits in recently burned habitats. Although fire‐driven changes in animal coloration and background matching have been reviewed broadly (Duarte et al. 2025), most work on this subject has focused on species that have distinct color morphs (light vs. dark morph) (Forsman et al. 2011; Potash et al. 2020), instead of animals that can physiologically change dorsal darkness. Furthermore, most prior work has neglected to quantify how animals are viewed through the visual systems of actual predators, nor how shifts in coloration affect the background matching of animals in burned areas (i.e., does dorsal darkness enhance background matching on burned substrates), a key component of crypsis.

Western fence lizards ( *Sceloporus occidentalis* ), small iguanian insectivorous lizards common throughout the western United States, are an ideal study system to test shifts in behavior and coloration in response to fire‐induced changes in prey vulnerability. They are a diurnal basking species that may be particularly vulnerable to reduced vegetation cover following fire, occur in fire‐prone habitats, are well known to survive large wildfires (i.e., they are found in burned areas immediately after fire; Rochester et al. 2010), and exhibit both morphological and physiological changes in dorsal darkness. The dorsal darkness of fence lizards, like many reptiles, is influenced by both morphological factors such as the relative abundance and arrangement of pigment cells and their association with melanin (Rosenblum 2005), and physiological factors including temperature (Langkilde and Boronow 2012) and hormonal regulation of melanin granule dispersion within skin cells (Hadley and Goldman 1969). Individuals can plastically shift dorsal darkness in response to temperature (Langkilde and Boronow 2012; Smith et al. 2016; Cadena et al. 2018) or circulating hormones (Hadley and Goldman 1969; Cox et al. 2008; Stepanek et al. 2019), but populations can also strongly differ in darkness as a response to ecological selection pressures (e.g., high elevation versus low elevation sites that differ in environmental conditions; Leaché et al. 2010). Consequently, post‐fire shifts in dorsal darkness may occur either through plasticity or selection. Prior work has documented potential fire‐adapted traits in fence lizards such as behaviors that help with detection of an oncoming wildfire (i.e., through sensory cues; Álvarez‐Ruiz et al. 2023), and changes in perching behavior and dorsal coloration in burned habitats. For instance, Lillywhite and North (1974) observed that while fence lizards typically perch on exposed rocks in unburned areas, they showed a preference for charred woody plants in burned habitats during the first 2 years post‐fire. Lizards from burned areas also exhibited increased dorsal darkness, which may enhance background matching on charred substrates (Lillywhite et al. 1977). In Western Fence Lizards, behavioral choices (e.g., selecting darker perches) and color change may act together to improve crypsis following fire, however this idea has not been formally tested using animal visual models.

We fill this information gap by studying fence lizards in an area that recently experienced a large wildfire. We quantified the behaviors, coloration, and background matching of western fence lizards to determine whether wildfire survivors exhibit traits that may enhance survival in burned environments. We predicted that detectability of lizards would be greater in burned areas due to reduced vegetation cover and lizards in burned areas would exhibit darker dorsal coloration and enhanced background matching on darker substrates compared to those in unburned habitats. Additionally, we evaluated perch selection behaviors to determine whether lizards choose perches that enhance their background matching in post‐fire environments where their vulnerability may be elevated. We predicted that lizards would prefer to use perches on which they are most cryptic, exhibiting greater color overlap with preferred perches compared to non‐preferred ones.

## 2 Materials and Methods

### 2.1 Study Sites

**FIGURE 1:**
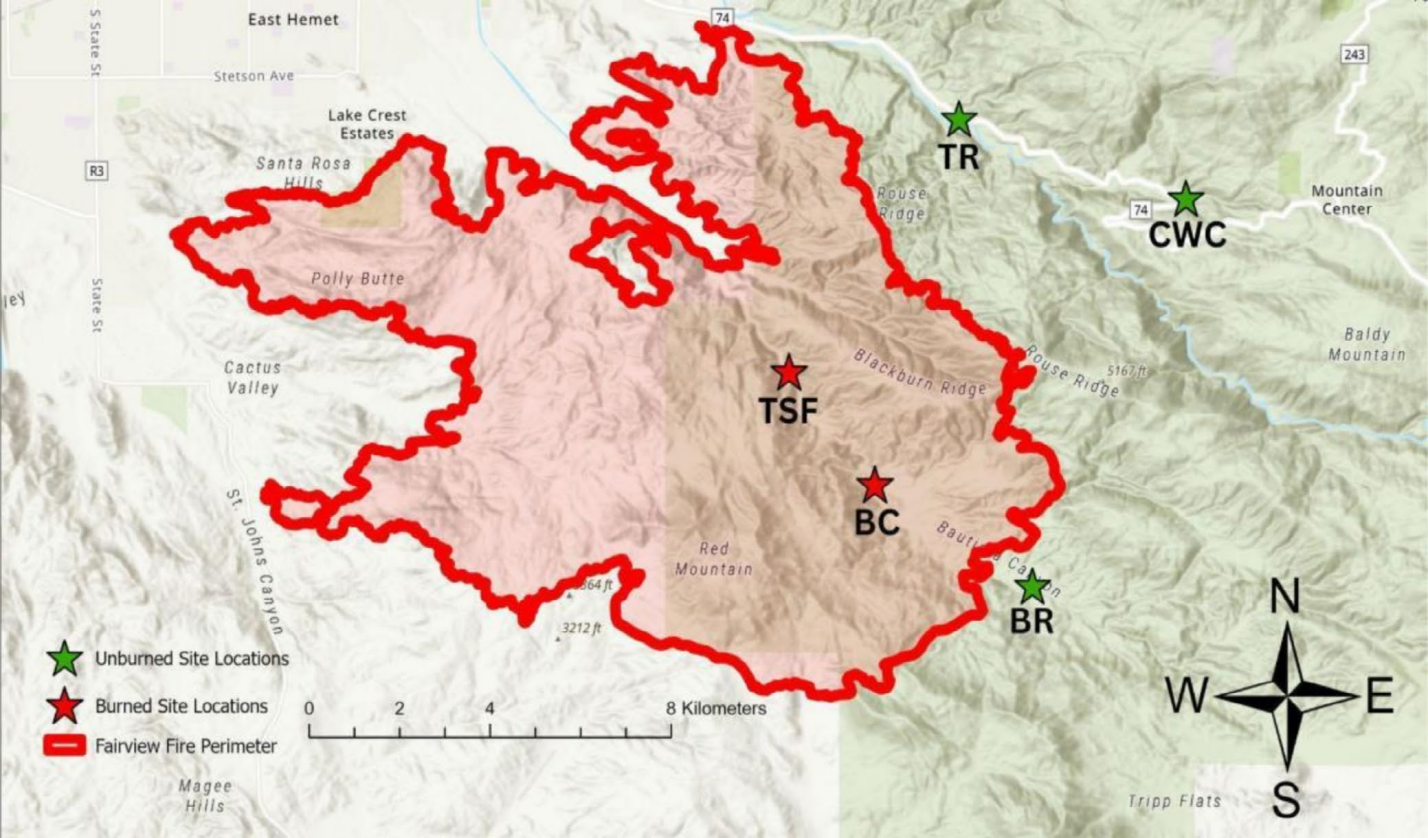
Burned and unburned study sites where data were collected. The red outline denotes the Fairview Fire perimeter. The green stars represent unburned sites: Toll Road (TR), Cold Water Creek (CWC), and Bautista Road (BR). The red stars represent burned sites: Two Stream Fork (TSF) and Baisley Creek (BC). Perch availability data were not collected in CWC due to accessibility issues.

This study was conducted from June to October 2023 along the eastern boundary of the San Jacinto Mountains near Hemet, California—a fire‐prone region dominated by shrubby chaparral vegetation and characterized by cool, wet winters and hot, arid summers. The area experienced the Fairview Fire in September 2022, which burned approximately 11,360 ha (Figure 1). Within the Fairview Fire burn scar, we surveyed two burned sites: Two Stream Fork (33.673426°, −116.834018°) and Baisley Creek (33.652000°, −116.816990°). For comparison, we surveyed three unburned sites: Toll Road (33.721571°, −116.800283°), Bautista Road (33.632678°, −116.785710°), and Cold Water Creek (33.706419°, −116.755247°). However, data collection at Cold Water Creek was limited due to restricted access.

None of the unburned sites had experienced fire in over 100 years. To avoid overlap in fence lizard populations, sites were spaced by at least 2 km. This distance exceeds the typical home range size for the species (mean 118.6 m^2^; Sheldahl and Martins 2000), making individual movements between sites unlikely, although gene flow may occur among these populations. Notably, burned and unburned sites were on average 6–8 km away from each other, ensuring distinct populations between these two habitat types.

### 2.2 Canopy Openness and Prey Vulnerability

To determine whether burned habitats are more open with less vegetation cover, we measured canopy openness through hemispherical photography. We used an Olympus TG‐6 digital camera equipped with a 180° fisheye lens and captured a total of 83 photos across both habitats (*N* = 36 in burned, *N* = 47 in unburned). Photos were taken at locations where western fence lizards were observed perching. The camera was positioned at lizard height and upward toward the sky. Photos of the canopy directly above were captured remotely using the OI.Share wireless application. Camera settings were standardized (ISO 800, F8.0, 1080p (fine) resolution), and images were processed using ImageJ with the Hemispherical 2.0 plugin to calculate gap fraction (Beckschäfer 2015). Gap fraction indicates the proportion of sky visible through the canopy, representing the extent of vegetation cover. Higher values indicate greater canopy openness. At each location, lizards were also captured and marked (detailed in Lizard Capture and Photography section below) to avoid duplicate observations of the same individuals. Thus, each photo represents a single perch location of a unique individual.

We quantified prey vulnerability using clay models of lizards. Artificial clay models are commonly used to quantify predation risk in various taxa, including lizards, because avian and mammalian predators leave visible attack marks in pliable clay (Wilgers and Horne 2007; Steffen 2009; Calderon‐Chalco and Putman 2019). To create clay models of western fence lizards, we first created silicon molds (Mold Star 16 Fast 1A:1B Volume Platinum Silicone Rubber) by immersing a 3D printed plastic model of a western fence lizard (snout‐vent length 75 mm) acquired from the 3D Operative Temperature Model Repository (https://www.3dotm.org/). Once the silicon molds were created, approximately 0.40 g of Sculpey III Hazelnut Clay was pressed into the mold to form a lizard model, and this was done repetitively to create the total number of models used in this study. We made 451 models of western fence lizards and the same number of 3 × 3 inch square control models to ensure that the focal animal models are attacked because they resemble actual prey, not due to the novelty of the item (Bateman et al. 2017; Rößler et al. 2018). All models were sprayed with a coat of gray textured paint (Rust‐Oleum textured spray paint in Aged Iron) to resemble the color of a western fence lizard through an avian predator visual system via the QCPA Framework detailed below (Van Den Berg et al. 2020).

Clay models were deployed along transects within burned and unburned sites during July (*N* = 106 in unburned, *N* = 106 in burned) and October 2023 (*N* = 120 in unburned, *N* = 120 in burned). Both lizard and control types were deployed across three burned sites and three unburned sites. Each deployment consisted of a set of lizard and control models placed within 1 m of each other, positioned on rock perches used by fence lizards. New pairs were placed every 5–10 m along the transect. We placed models on light‐colored rock substrates to standardize the background as much as possible (rocks are widespread and common across both habitats unlike other types of substrates) and to enhance their visibility to predators as prior work report low attack rates on large sample sizes of models (Bateman et al. 2017; Paluh et al. 2015; Calderon‐Chalco and Putman 2019). In July, models were removed after 48 h (Watson et al. 2012) because the heat started to harden the clay after this time. In October, we left models out for 1 week and checked them in 48 h increments (Shepard 2007; Calderon‐Chalco and Putman 2019). Predation marks (i.e., scratch/bite marks) were recorded as evidence of predator attacks, however, marks were not classified by predator type because imprints were often ambiguous and could not be reliably assigned to birds versus mammals specific predators. Models that were missing from their original positions were noted but not included in statistical analyses as we could not confirm whether they were missing due to predators or other factors. Missing or attacked models were not replaced.

### 2.3 Lizard Capture and Photography

To quantify dorsal darkness and background‐matching of western fence lizards, we photographed captured individuals in the field. A total of 37 adult lizards from burned sites and 38 from unburned sites were photographed during June and July 2023. Sites were visited in a balanced design to reduce order effects. We looked for lizards from morning through early afternoon by slowly walking through each site and scanning potential perch locations. Lizards were captured using an extendable pole with a slipknot at the end. Immediately upon capture, lizards were positioned dorsal side up on a white background and a photograph was taken of them. We used an Olympus TG‐6 camera mounted on a tripod 15 cm overhead, leveled using the built‐in level. Camera settings followed Wuthrich et al. (2022): RAW format, aperture priority mode, ISO 800, f/8.0, and 1080p (fine) resolution. Each photograph included an 18% gray card and a scale bar for standardization, and a black umbrella was held overhead to shade the field of view and minimize lighting variation.

To control for potential effects of color change over time due to stress (Lewis et al. 2017; Stepanek et al. 2019), we recorded the time from the moment the lizard was first disturbed (i.e., defined as when it moved from its original perch location during capture attempts) until the photograph was taken, using a stopwatch. All lizards were photographed within 5 min of pursuit, and we evaluated the effect of time to photo in the analyses below. We also recorded each lizard's body temperature using an infrared temperature gun (EnnoLogic et650D) as dorsal darkness may correlate with temperature. Additional biotic measurements included body size as the snout‐to‐vent length (SVL), mass, evidence of prior tail autotomy and sex. Notably, tail autotomy is often used as a proxy for predation pressure, but it has limitations because tail loss can result from multiple factors and may reflect predator inefficiency rather than true predation risk (Medel et al. 1988). In our study, tail autotomy could not be used as a reliable indicator of risk due to wildfires because we could not determine whether tail loss occurred before or after the fire, making direct comparisons between burned and unburned populations infeasible. After processing, lizards were marked with non‐toxic paint to prevent recapture of individuals and released at their place of capture. All work with lizards was performed under a California State University, San Bernardino IACUC approved protocol (# 19‐019) and California Department of Fish and Wildlife scientific collecting permit (S‐192820001‐19282‐001).

For background matching analyses, photos were taken of the substrate on which the lizard was initially found, approximately 30 cm from the substrate surface. We used the same camera and lighting conditions as those used for the lizard photography detailed above and included the 18% gray card, along with a scale bar, for post‐processing analyses.

### 2.4 Dorsal Darkness and Background Matching

We used the micaToolbox plugin in ImageJ (Troscianko and Stevens 2015) to quantify lizard dorsal darkness and background matching through the visual properties of an avian predator. Prior to photo analyses, we created a cone‐catch model, which converts images taken by a specific camera to the spectral sensitivities of a given visual system. We used a standard color chart with known spectral reflectance curves (Xrite/Calibrite Mini Colorchecker) to create the cone catch model for the bluetit avian visual system. We chose the bluetit because it is the closest relative of fence lizard avian predators (corvids and raptors) for which spectral sensitivities are well documented. For the cone catch model, we also only selected a wavelength range of 400–700 nm because prior work on fence lizards has documented little to no ultraviolet reflection off their dorsal surfaces (i.e., what would be visible to diurnal predators) (Putman et al. 2017). Once the cone‐catch model was made, for each RAW image file of each lizard, we created a calibrated multispectral image using the 18% gray standard within the photo. A calibrated image converts a non‐linear photo (meaning the pixel values do not scale linearly with light intensity) to a linear format, and controls for differences in lighting intensity and color between images, using the gray standard. After we made a multispectral image, we used the line tool in ImageJ to indicate the length of the scale bar in the photograph and saved this as a “region of interest” (ROI). The body and head region of each lizard was also outlined (excluding limbs and tail) using the polygon tool and saved as an ROI for further analyses. We quantified lizard dorsal darkness by calculating a single luminance value per individual, defined as the mean luminance measured across the lizard body and head ROI. A higher luminance value indicates that the lizard is brighter, and a lower value indicates that the lizard is darker, thus dorsal darkness is inversely related to luminance.

**FIGURE 2:**
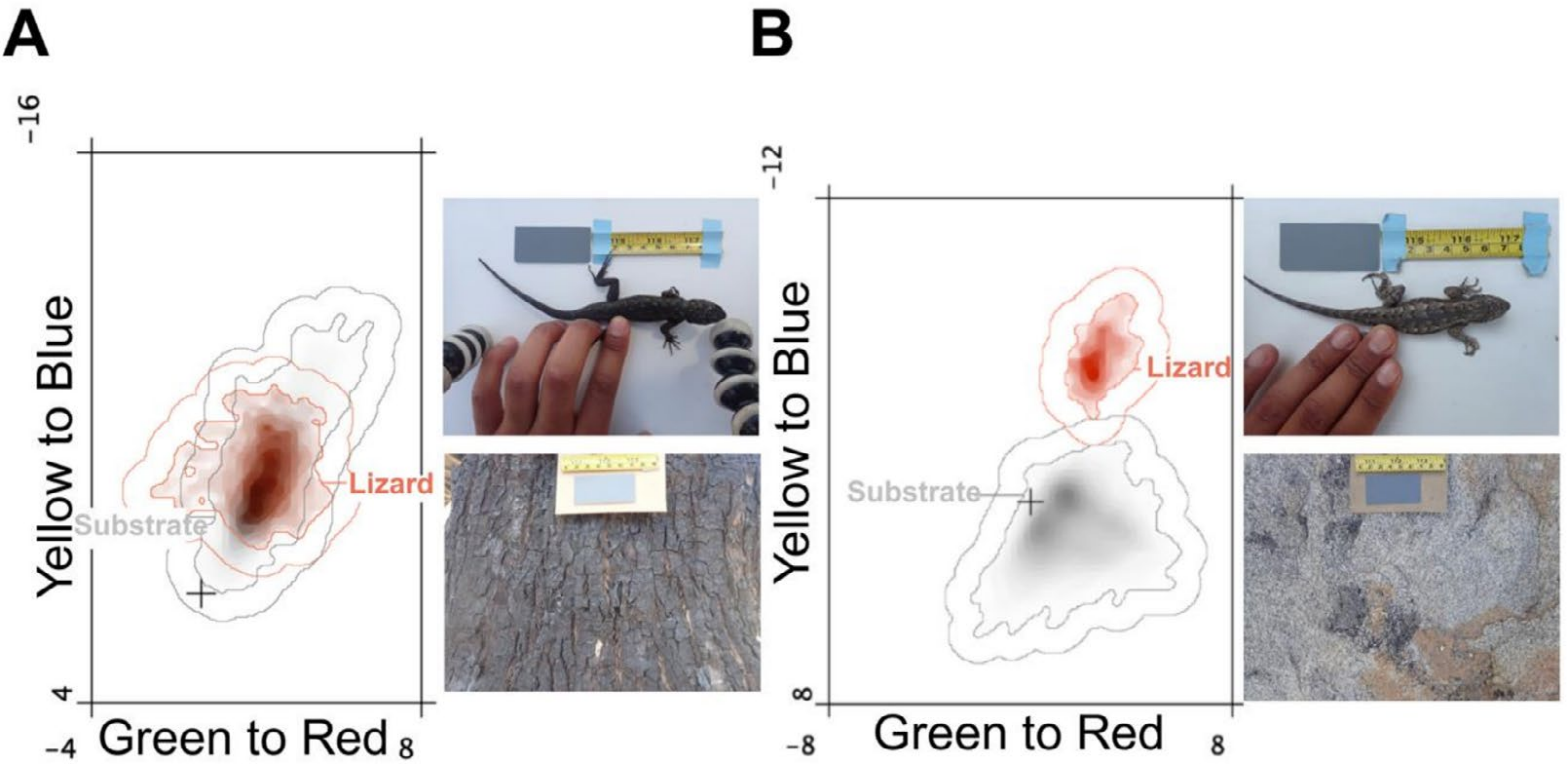
Color maps illustrating the dorsal region of interest for individual lizards (red point clouds) and their corresponding substrates (gray point clouds). The *X*‐axis represents variation from green‐red wavelengths, and the *Y*‐axis represents variation from yellow–blue wavelengths quantified within Receptor Noise Limited model chromaticity space. (A) Lizard from a burned site showing strong overlap with a burned wooden substrate. (B) Lizard from an unburned site demonstrating poor overlap against an unburned rock substrate.

We used the Quantitative Color and Pattern Analysis (QCPA) through the micaToolbox plugin (Van Den Berg et al. 2020) to determine how each lizard's color is portrayed against its background. This analysis was applied to all lizard multispectral images and their respective substrate photos (which were also converted to calibrated multispectral images using the cone catch model detailed above). When running the QCPA, we applied an acuity correction, which controls for the avian receiver's spatial acuity and viewing distance, and the Gaussian acuity control correction, which is recommended for non‐rectangular ROIs. We also applied a receptor noise limited ranked filter, which performs noise reduction while preserving chromatic and luminance edges. We set the luminance Weber fraction to 0.1 and the acuity units (cycle per degree) to 30, which represents an avian predator species of fence lizards (specifically for corvids; Fite and Rosenfield‐Wessels 1975). The viewing distance was set at 1240 mm and was determined using the mean flight initiation distance of western fence lizards (S. Ruck, unpublished data). Receptor Noise Limited (RNL) color maps of each ROI (lizard and substrate) were created, which are gray scale intensity images showing the distribution of pixels in the log transformed RNL color space with the intensity representing the amount of pixels in an ROI sharing the same coordinates. We plotted and compared RNL color maps of the lizard and its substrate to calculate their overlap in color space. The extent of overlap between the two maps correlates with the degree of background matching between the lizard and its corresponding substrate, with higher values indicating a greater level of camouflage than lower values (Figure 2).

### 2.5 Perch Preferences in Burned Sites

To evaluate whether lizards prefer to use perches that associate with higher levels of background matching, we compared perch availability in the habitat to perch use by lizards. To estimate perch availability, we conducted 15 randomized 20‐m transects in each habitat type during the same months that we captured and photographed lizards. At every meter, we stopped and scanned a meter to the left and to the right and noted every rock or wooden perch (Bors et al. 2020) available from 10 cm to 3 m in height encompassing the range of perch heights used by fence lizards (Zani 2001). Perches were categorized as burned or unburned rock and wood, with burned substrates identified by > 50% charred surface coloration.

### 2.6 Statistical Analyses

Statistical analyses were conducted in R 4.4.1 (R Core Team 2023) using mixed‐effects models (through the lme4 package; Bates et al. 2015) with site (5–6 levels) always included as a random effect to account for non‐independence of observations within sites. Although the number of sites was modest, site was not a focal predictor, and random‐effects models showed no evidence of singular fits or convergence issues, supporting its use for controlling site‐level variation. Model assumptions were met through examination of residual plots. In all models, we tested for interactions among predictors and if interaction terms were not significant, they were removed from models and only main effects were assessed. Post hoc analyses were performed using the emmeans package (Lenth 2024).

Canopy openness (i.e., gap fraction) was analyzed with a linear mixed‐effects model with habitat type as the fixed effect. Attacks on clay models were analyzed using a binomial model including confirmed attacks (yes or no) as the response variable, habitat type (burned vs. unburned), survey month (July or October), and model type (lizard or control) as fixed effects.

To test whether lizard dorsal darkness and background matching were affected by habitat and/or the background environment, we used two separate linear mixed‐effects models that each included habitat type and substrate luminance as the fixed effects, and luminance (inversely related to darkness) or level of background matching (i.e., percent overlap of color maps) as the response variable. Lizard sex (*χ* ^2^ = 3.050, df = 1, *p* = 0.081), body temperature (*χ* ^2^ = 1.767, df = 1, *p* = 0.184), and time to photo from capture (*χ* ^2^ = 0.395, df = 1, *p* = 0.530) did not significantly affect dorsal luminance. Similarly, sex (*χ* ^2^ = 2.457, df = 1, *p* = 0.117), body temperature (*χ* ^2^ = 1.652, df = 1, *p* = 0.199), and time to photo (*χ* ^2^ = 0.034, df = 1, *p* = 0.854) did not significantly affect background matching (percent overlap). Thus, these variables were not included in the final models. To evaluate whether different perch types (burned, wood, rock) varied in luminance, we ran a separate linear mixed‐effects model with perch type as the fixed effect and substrate luminance (log‐transformed) as the response variable. Finally, we used Chi‐squared tests to compare observed perch use by lizards to what would be expected based on the availability of perches in the environment estimated from the transect surveys.

## 3 Results

### 3.1 Canopy Openness and Prey Vulnerability

**FIGURE 3:**
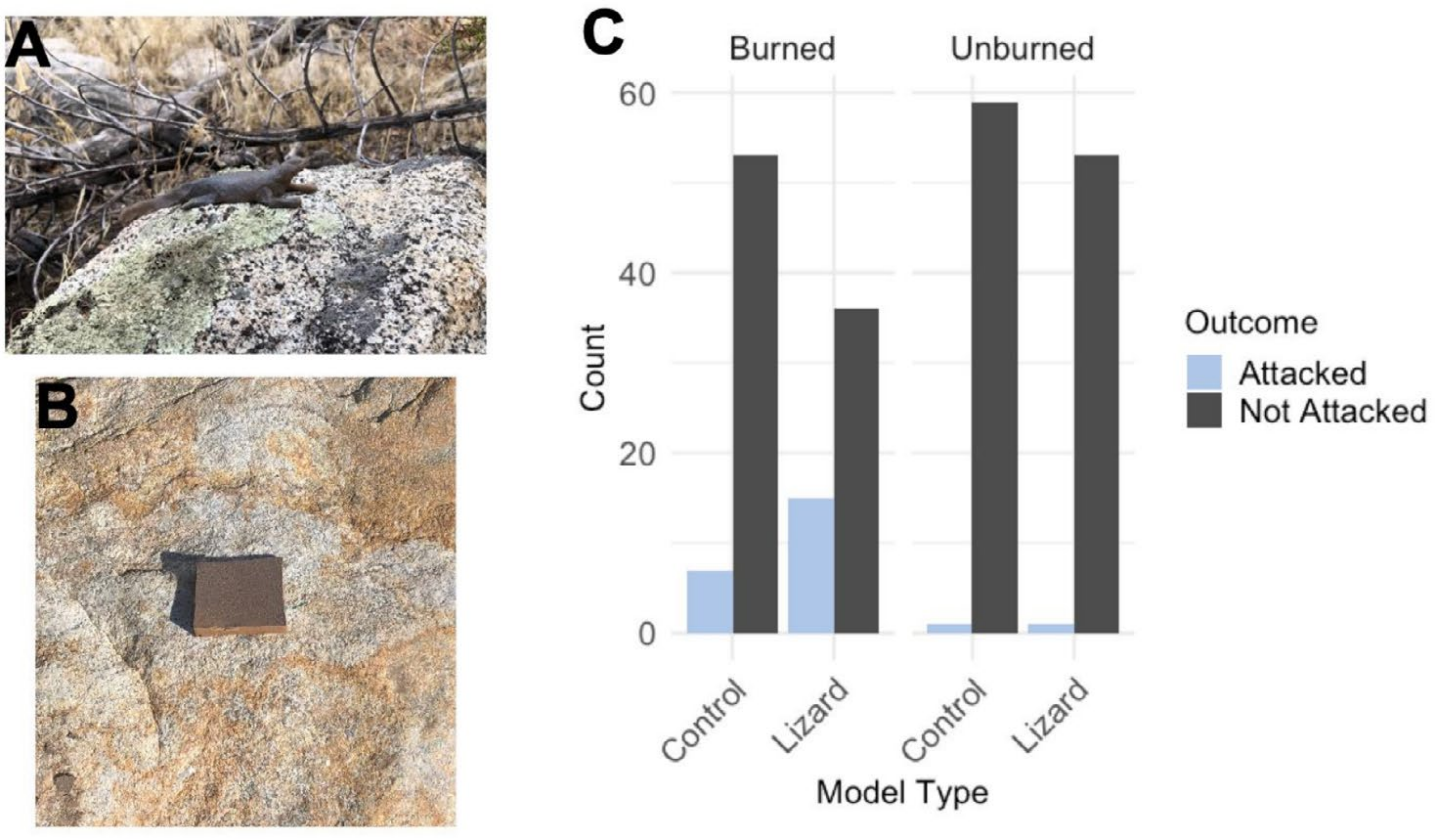
(A) Clay lizard model. (B) Control model. (C) Number of models with confirmed attacks only for the month of October.

Burned habitats had greater canopy openness (measured as gap fraction) than unburned habitats (*χ* ^2^ = 6.0266, df = 1, *p* = 0.014). For prey vulnerability, we found a significant interaction between habitat type and month on the proportion of clay models attacked (*χ* ^2^ = 10.012, df = 1, *p* = 0.002). In July, there was not a significant difference in attacks between burned and unburned habitats (*p* = 0.889), but in October, attack rates were significantly higher in burned habitats (*p* = 0.021, Figure 3). There was also no significant difference in predator attacks between the different model types (lizard and control) (*χ* ^2^ = 1.868, df = 1, *p* = 0.172).

### 3.2 Lizard Dorsal Darkness and Background Matching

**FIGURE 4:**
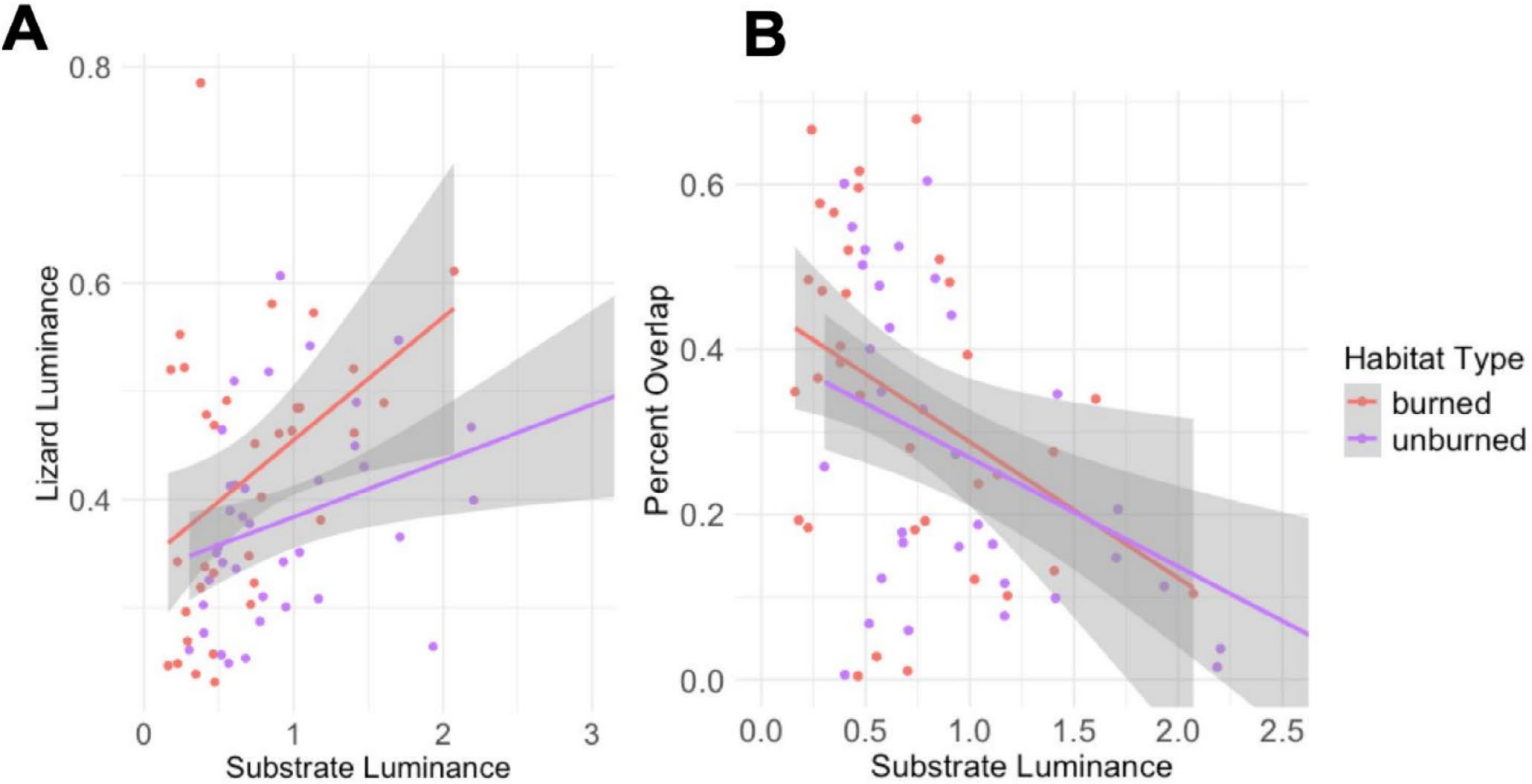
(A) Positive relationship between lizard luminance and substrate luminance across burned and unburned habitats. (B) Negative relationship between substrate luminance and background matching between lizard and its background across burned and unburned habitats. In both panels, lines represent linear model fits and shaded regions depict 95% confidence intervals. An observation with a substrate luminance of 4.0 was excluded from this figure for visual clarity.

Lizards were darker and better matched on darker substrates (e.g., burned substrates) than lighter substrates. Substrate luminance had a significant positive effect on lizard luminance (*χ* ^2^ = 11.163, df = 1, *p* < 0.001, Figure 4A) and a negative effect on their background matching (*χ* ^2^ = 16.555, df = 1, *p* < 0.001, Figure 4B). Burned substrates had much lower luminance values than unburned substrates (*χ* ^2^ = 86.855, df = 2, *p* < 0.001, burned vs. wood: *p* < 0.0001, burned vs. rock: p < 0.0001), demonstrating that they are the darkest substrate type available to lizards. Between unburned substrates, wood was darker than rock (wood vs. rock: *p* = 0.004). Although burned substrates were widely available for use in burned habitats (see below for results), lizards captured in burned sites were not significantly darker (*χ* ^2^ = 3.404, df = 1, *p* = 0.065) or better background‐matched (*χ* ^2^ = 0.360, df = 1, *p* = 0.549) than lizards from unburned sites. Conversely, the non‐significant effect on dorsal darkness reflected a potential trend toward higher luminance (i.e., lighter coloration) in lizards from burned habitats compared to unburned habitats (Figure 4A).

### 3.3 Perch Preferences

**FIGURE 5:**
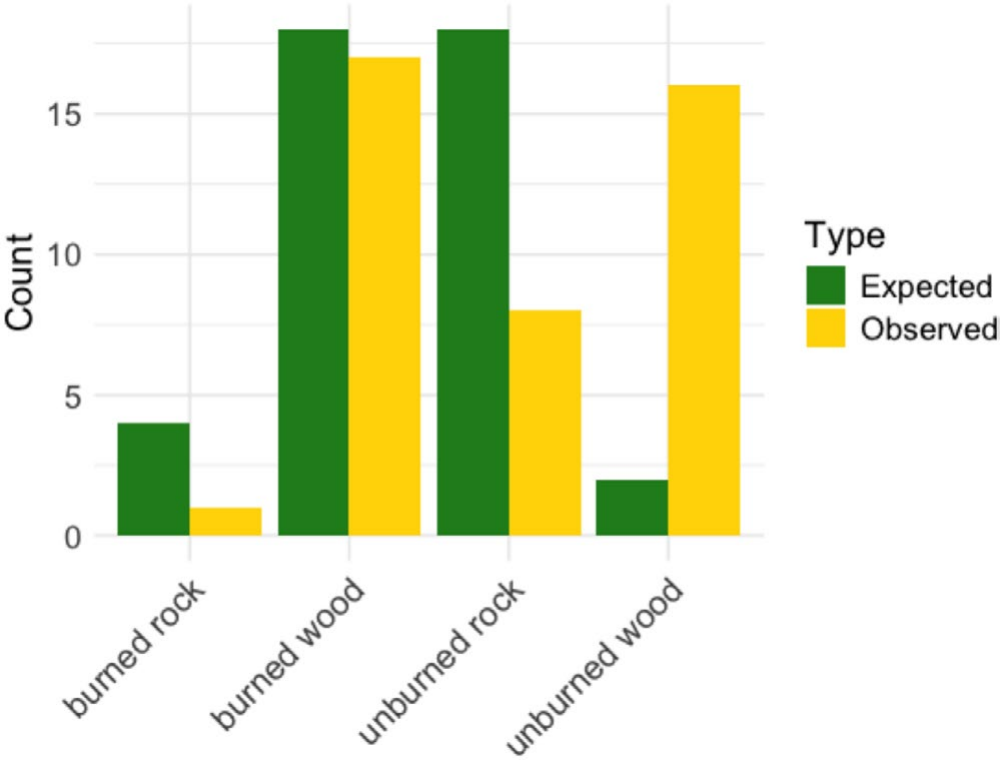
Expected and observed lizard perch use within burned habitat.

Lizards in both burned and unburned habitats significantly preferred to use wood perches over rock perches, despite the higher abundance of rocks in the surrounding environment (unburned: *χ* ^2^ = 28.490, df = 1, *p* < 0.0001; burned: *χ* ^2^ = 106.909, df = 1, *p* < 0.0001). Within burned sites, lizards' observed use of perches greatly differed from expected based on quantified perch availability (*χ* ^2^ = 105.861, df = 3, *p* < 0.0001, Figure 5). Lizards showed a clear preference for using unburned wood and an avoidance of rocks.

## 4 Discussion

We found evidence that fires decrease canopy cover, which may increase the vulnerability of lizards and other animals. However, fence lizards did not seem to modify perch selection behaviors in burned habitats to enhance their camouflage in this altered environment. Instead, lizards preferred to use unburned wood perches, even though lizards blend more effectively with the darkened, charred substrates found in burned areas. While lizards preferred unburned wood, they were still frequently observed on burned substrates, likely due to the abundance of burned wood in these habitats. In such cases, their enhanced camouflage may reflect adaptation to altered post‐fire environments. However, western fence lizards may be affected by trade‐offs (e.g., with thermoregulation) associated with using these perches because they showed a strong preference for unburned perches. As fires become more frequent and severe, animals like western fence lizards may be forced to use more darkened substrates in post‐fire environments, which may increase heat exposure and potentially affect reproduction (Domínguez‐Godoy et al. 2024) or survival (Abram et al. 2017).

We found that recent fire may increase prey vulnerability in burned habitats which may result in mortality through predation. By placing artificial clay models, we observed an increase in attacks at the burned sites, which also had reduced vegetation cover. This suggests that post‐fire landscapes may expose wildlife to heightened risk with predators potentially benefiting from the altered environment (Leahy et al. 2016; Hovick et al. 2017; Bonta et al. 2017; Cherry et al. 2017). Several factors may increase prey vulnerability after fires. First, post‐fire changes in habitat structure can lead to greater exposure making prey more visible to predators (Leahy et al. 2016; Hradsky et al. 2017). Our canopy cover results support this, showing that burned areas had less cover, presumably making lizards more visible to roving predators. This aligns with other studies that demonstrate increased predation post‐fire due to greater visibility caused by reduced vegetation cover (Leahy et al. 2016; Hovick et al. 2017; Bonta et al. 2017). Increased risk in burned areas may also result from shifts in predator behavior. If predators are attracted to recently burned areas, predation rates can increase due to the higher density or diversity of predators in these spaces. For instance, the Texas horned lizard ( *Phrynosoma cornutum* ) is attracted to burned areas where the abundance of its prey, harvester ants (*Pogonomyrmex* spp.), increases post‐fire. Similarly, even as vegetation begins to regrow, predation rates may remain elevated as herbivores are drawn to these areas to feed on the regenerating vegetation, thus attracting predators (Green et al. 2015).

The significant interaction between habitat type and month on clay model attacks may suggest that predation pressure varies seasonally (Ferreira and Faria 2021). For example, Lister and Aguayo (1992) reported a 19% increase in predatory attacks on clouded anole ( *Anolis nebulosus* ) clay models during the dry season compared to the wet season in Mexico. However, it is more likely that the duration that the models were left out influenced the non‐significant difference in predation attacks between burned and unburned habitats in July. In July, we could only leave models out for 48 h until they hardened due to the intense heat of the summer. In October, models were left out for a full week and this extended exposure may have allowed predators more time to acclimate to the models, a factor noted in previous studies (Shepard 2007; Calderon‐Chalco and Putman 2019). Regarding clay model types, predators did not show a strong preference for attacking lizard models over control models. This was surprising considering the attention to detail we took when making the lizard models (i.e., color, shape, size, positioning), and previous studies' results showing predators distinguishing between prey and control models (Mason et al. 2018; Orton et al. 2018; Velo‐Antón and Cordero‐Rivera 2011; Paluh et al. 2015). The accuracy of the models' coloration to that of actual prey may play a crucial role in directing predator attacks, especially for avian predators (Marshall et al. 2016). Both model types were painted to match the color of western fence lizards, verified through comparisons with photographs of live lizards (run through an avian visual model). On the other hand, if models were attacked by predators that rely on olfactory cues (e.g., small mammals), it is possible that the novelty of the clay's scent induced curiosity‐driven attacks (Pärt and Wretenberg 2002). However, it is also important to note that small mammals (i.e., rodents) have been documented consuming lizards, thus some attacks may reflect legitimate predatory behavior (Norbury et al. 2014; López‐Darias et al. 2024). Because predator identity could not be reliably determined and overall sample sizes were low, we could not test the effects of predator type statistically. Nonetheless, regardless of whether models were attacked due to legitimate predation attempts or predator curiosity, those placed in burned habitats were more likely to suffer damage, suggesting increased detectability and vulnerability in these environments. This heightened exposure may translate to elevated predation risk for real animals.

Contrary to our expectations, lizards that recently survived a large wildfire did not exhibit significantly darker dorsal surfaces compared to lizards in unburned habitats (quantified as luminance). This result contrasts with previous studies showing increased dorsal darkness in animals occupying burned habitats (de Alcantara Viana et al. 2022; Potash et al. 2020; Lillywhite et al. 1977; De Miranda et al. 2022). For example, *Norops meridionalis* lizards are darker in coloration in post‐fire areas, likely as a form of “pyrogenic camouflage” (De Miranda et al. 2022). Similarly, de Alcantara Viana et al. (2022) showed that various arthropods occupying recently burned savannas exhibited background matching behaviors, selecting darker backgrounds to reduce contrast with their body coloration. In our study, however, lizard dorsal darkness was more strongly driven by the luminance of the substrate than by fire history. This suggests that perch use or selection may play a larger role in achieving crypsis than morphological change alone. Our results demonstrate that lizards are darker on burned backgrounds (which only occur in the burned habitats) because burned perches were significantly darker than unburned perches, and lizard dorsal darkness significantly correlated with perch darkness. However, because lizards did not prefer to use burned perches, the overall lizard population was not darker than that in the unburned habitat. Thus, lizard dorsal darkness in recently burned habitats seems more like a plastic response to substrate selection than a result of selection for darkened dorsal morphology (i.e., non‐random survival of darker individuals), but further studies using an experimental approach are needed to tease apart the role of phenotypic plasticity from genetic adaptations in these responses.

We also found that lizards' background matching increased with substrate darkness, suggesting greater camouflage in burned habitats. Incorporating avian visual models allowed us to quantify camouflage from the predator's perspective, providing more ecologically realistic assessments of concealment effectiveness (Mark et al. 2022; Walton and Stevens 2018). Without accounting for predator vision, conclusions about background matching and crypsis may misrepresent real‐world survival dynamics. Our study contributes to a growing body of work examining background matching in post‐fire environments by applying predator visual models to fire‐altered substrates (de Alcantara Viana et al. 2024). However, as above, further research that tests lizard populations from habitats of varying fire regimes and that experimentally alters background environments are necessary to determine whether dorsal darkness and enhanced matching on darkened substrates is in fact an adaption to wildfires.

Although lizards exhibited better background matching on darker, burned substrates, they did not preferentially use burned perches within burned habitats. Lizards used burned wooden perches at nearly the same level at which they were available. However, lizards exhibited a strong perch preference for unburned wood, which was a limiting resource in burned habitats. This pattern mirrors recent findings in a praying mantis (*Mantis religosa*), where melanic and brown morphs differed in camouflage effectiveness on burned versus unburned tree trunks, yet they consistently preferred unburned substrates when given a choice (de Alcantara Viana et al. 2024). One explanation may lie in the trade‐offs faced by animals in burned environments. While dark perches may offer superior camouflage, they may also retain more heat, leading to potential thermal stress, especially during the hotter summer months when our study was conducted. During field data collection, ambient temperatures at our sites regularly exceeded 38°C (personal observation). In ectothermic species, external coloration can also affect thermoregulation, with darker dorsal pigmentation increasing the absorption of radiant energy (Trullas et al. 2007; Broennimann et al. 2014). Lizards on darkened substrates must be darker for effective crypsis, but at the cost of increasing their own internal temperature. Temperature constraints can have negative short‐ and long‐term consequences for ectothermic animals, like lizards (Zhang et al. 2018; Abram et al. 2017). Lizards can experience long‐term population declines due to chronic heat exposure. Thermal constraints on activity reduce their ability to forage, grow, and reproduce (Domínguez‐Godoy et al. 2024). Therefore, unburned wood may provide a cooler microhabitat (Yan et al. 2019; Lee and Jim 2018) that allows lizards to remain active while reducing the risk of overheating. Interestingly, we also found a non‐significant trend suggesting that lizards in burned habitats had higher dorsal luminance, indicating they were brighter, not darker, than those from unburned habitats. One possible explanation is that increased brightness may help mitigate overheating in high‐temperature environments (Cadena et al. 2018; Geen and Johnston 2014; Smith et al. 2016) and may be more advantageous than dorsal darkness in burned areas that have high ambient temperatures, although further research is needed to evaluate this hypothesis.

Furthermore, fire may impose multiple behavioral trade‐offs that extend beyond thermal constraints, potentially explaining why fence lizards strongly preferred unburned wood in burned habitats. Locomotor performance is a key determinant of survival (Bennett and Huey 1990), and substrate characteristics can influence sprint speed and maneuverability (Battles et al. 2019; Savvides et al. 2019). Because some lizards perform better on rough or woody substrates (Tulli et al. 2009), while others achieve higher performance on smoother surfaces (Vanhooydonck et al. 2005), charred substrates may alter escape efficiency or foraging success in western fence lizards. Chemical deposits on charred surfaces or ash may also interfere with chemosensory communication; for instance Howey and Snyder (2020) found that newborn timber rattlesnakes ( *Crotalus horridus* ) had reduced ability to follow maternal scent trails on burned ash compared to unburned substrates. Additionally, fire‐induced changes to invertebrate prey communities may influence microhabitat use as prey availability can vary depending on burn severity and intensity (Schmitz et al. 2015; Hansen 1986). Some lizard species may benefit from wildfire through increased prey availability (Griffiths and Christian 1996; Hellgren et al. 2010). However, these responses are highly species‐specific, and not all lizards experience positive outcomes following fire disturbance (see review Santos, Sitters, et al. 2022). Together, these studies suggest that perch selection behavior in post‐fire environments likely reflects multiple interacting factors. Furthermore, wildfires vary in severity, intensity, and extent and these factors, including rates of post‐fire succession likely all interact to influence animal behavioral responses to fire. Our study focused on a single large wildfire in a single mountain range in Southern California, therefore our results may not be generalized to all animals living in all post‐fire environments. This highlights the need for studies across multiple fire‐prone regions at various stages of fire recovery to generate a comprehensive understanding of organismal responses to wildfires, information that will be necessary for species conservation in an increasingly flammable world.

## 5 Conclusions

The potential for more frequent and intense wildfires is increasing as climate change drives severe droughts and extreme heat events, and anthropogenetic activities increase ignition sources. The need to understand the consequences and implications of fires on animals and their behavior is critical. In this study, we found compelling evidence that fires increased canopy openness, which may increase the vulnerability of prey in burned areas. We also found that western fence lizards may be capable of effective background‐matching on burned substrates in post‐fire environments but they do not prefer to use these substrates. Instead, lizards showed a strong preference for unburned wood perches. As fires become more frequent and severe, animals like western fence lizards may be forced to use more darkened substrates in post‐fire environments, but their reproduction and survival may be affected by trade‐offs associated with using these substrates (e.g., impacts to thermoregulation). Overall, we shed light on how wildlife might adapt to the drastic habitat changes caused by anthropogenic‐induced wildfires.

## Author Contributions

**Elmer E. Gutierrez:** data curation (lead), formal analysis (equal), investigation (lead), methodology (equal), writing – original draft (lead). **Breanna J. Putman:** data curation (supporting), formal analysis (equal), funding acquisition (lead), investigation (supporting), methodology (equal), supervision (lead), writing – review and editing (lead).

## Funding

This work was supported by Office of Student Research CSU San Bernardino funded for field data collection assistants.

## Conflicts of Interest

The authors declare no conflicts of interest.

## Supporting information


**Data S1:** ece373673‐sup‐0001‐Supinfo.zip

## Data Availability

Data, metadata, and code are openly available at Dryad: https://doi.org/10.5061/dryad.d51c5b0jz.
